# Complete Loss of Cramp Promotes Experimental Osteoarthritis with Enhanced Chondrocyte Apoptosis in Mice

**DOI:** 10.3390/ijms26167874

**Published:** 2025-08-15

**Authors:** Moon-Chang Choi, Jiwon Jo, Junghee Park

**Affiliations:** 1 Department of Biochemistry and Molecular Biology, School of Medicine, Chosun University, Gwangju 61452, Republic of Korea; 2 Department of Cellular and Molecular Medicine, School of Medicine, Chosun University, Gwangju 61452, Republic of Korea; ehklwl55@chosun.kr; 3 Institute of Well-Aging Medicare & Chosun University G-LAMP Project Group, Chosun University, Gwangju 61452, Republic of Korea

**Keywords:** osteoarthritis, cramp, cathelicidin-related antimicrobial peptide, cartilage degeneration, synovitis

## Abstract

Osteoarthritis (OA) is the most prevalent form of joint arthritis, frequently associated with aging, mechanical wear, and inflammation. Our previous work demonstrated that cathelicidin-related antimicrobial peptide (Cramp) is upregulated in mouse OA cartilage, and that transient knockdown (KD) of Cramp in cultured chondrocytes decreases IL-1β-induced expression of matrix-degrading enzymes. The aim of this study was to determine the in vivo role of Cramp in OA pathogenesis using whole-body Cramp knockout (KO) mice. Normal skeletal development and growth plate morphology were assessed in E18.5d embryos and 2-week-old mice, respectively. Expression profiles of catabolic and anabolic genes were analyzed in primary chondrocytes derived from Cramp KO mice. OA in mouse knee joints was induced using intra-articular monosodium iodoacetate (MIA) injections or surgical destabilization of the medial meniscus (DMM). We observed that Cramp loss does not impact normal skeletal development. In contrast to our expectations, complete Cramp deficiency in chondrocytes failed to decrease catabolic gene expression upon IL-1β stimulation. Instead, genetic deletion of Cramp significantly worsened OA cartilage degradation in both MIA- and DMM-induced models. The detrimental phenotype observed in Cramp-deficient mice results from enhanced chondrocyte apoptosis. Therefore, even minimal Cramp expression appears essential for maintaining catabolic balance and preventing chondrocyte apoptosis in OA cartilage. Collectively, our data indicate that Cramp may exert multifaceted effects on OA pathogenesis by modulating catabolic pathways and apoptosis.

## 1. Introduction

Osteoarthritis (OA) is a comprehensive joint disorder primarily resulting from aging [1]. OA-affected joints are characterized by articular cartilage degeneration, synovial inflammation, subchondral bone sclerosis, and osteophyte formation [1]. A hallmark of OA is the degradation of the cartilage extracellular matrix (ECM) mediated by either chondrocyte catabolism or apoptosis. Chondrocytes are capable of synthesizing ECM proteins to maintain cartilage homeostasis or generating matrix-degrading enzymes that promote cartilage breakdown. In fact, cartilage in OA, which is compromised by mechanical stress and synovial inflammation, exhibits pronounced ECM degradation due to the upregulation of catabolic factors [2,3]. Among the various matrix metalloproteinases (MMPs) and aggrecanases (ADAMTSs) that are activated by OA-associated stimuli, MMP-13 and ADAMTS5 have been identified as essential mediators of OA cartilage destruction [4,5]. Impaired chondrocyte survival further contributes to cartilage degeneration [6,7], as ECM-expressing chondrocytes are the exclusive cell type present in cartilage. The molecular mechanisms that derive chondrocytes to become ECM-catabolizing or apoptotic cells in OA development remain to be fully elucidated.

Cathelicidins are classified as host defense peptides that play a crucial role in protecting against microbial infections [8]. Mouse cathelicidin-related antimicrobial peptide (Cramp) and its human homolog LL-37 exhibit strong antimicrobial activity, and are produced and secreted by a variety of cell types, including neutrophils, macrophages, epithelial cells, and adipocytes [9,10,11,12,13]. While Cramp has been the subject of extensive research in the context of infectious diseases, recent studies have shown that Cramp also regulates tissue degeneration, including skeletal muscle, liver, and kidney, during non-infectious diseases, acting as either a disease-promoting or protective factor [14,15,16,17]. Our previous work suggested a causative involvement of Cramp in OA pathogenesis [18], as small interfering RNA (siRNA) knockdown (KD) of Cramp in chondrocyte cultures led to reduced expression of catabolic enzymes, and intra-articular injection of synthetic Cramp into mouse knee joints exacerbated experimental OA [18]. Nevertheless, whether in vivo inactivation of Cramp influences OA development remains unclear.

In this study, we utilized Cramp-deficient mice to assess the in vivo relevance of Cramp in OA pathogenesis. Unexpectedly, our results indicate that complete loss of Cramp increases susceptibility to experimentally induced OA injury. These findings highlight the complex and multifaceted roles of Cramp in OA pathogenesis.

## 2. Results

### 2.1. Cramp Knockout (KO) Mice Exhibit Normal Skeletal Development

**Figure 1:**
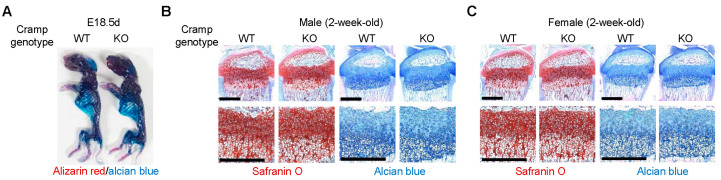
Cramp deficiency does not impact embryonic skeletal formation or post-natal growth plate development. (**A**) Skeletal staining of E18.5 embryos from Cramp knockout (KO) mice and their wild type (WT) littermates. (**B**,**C**) Safranin-O and alcian blue staining of growth plates from 2-week-old male (**B**) or female mice (**C**). Bars = 500 μm.

To investigate the in vivo function of Cramp in OA disease, we initially assessed whether Cramp inactivation affects normal skeletal development in mice. To this end, whole mouse embryos at E18.5d from Cramp wild type (WT) and KO mice underwent alcian blue and alizarin red staining (Figure 1A). The staining results indicated that Cramp KO embryos did not display detectable skeletal abnormalities, suggesting that embryonic skeletal development is unaffected by the absence of Cramp. Furthermore, assessment of 2-week-old mice after birth showed that Cramp KO did not alter growth plate architecture in either male or female mice, as evidenced by both safranin-O and alcian blue staining (Figure 1B,C). These data demonstrate that Cramp is dispensable for embryonic skeletal development and for post-natal growth plate formation under normal, unchallenged conditions.

### 2.2. Cramp-Deficient Chondrocytes Exhibit Partial Upregulation of Matrix-Degrading Enzymes Following IL-1β Stimulation

**Figure 2:**
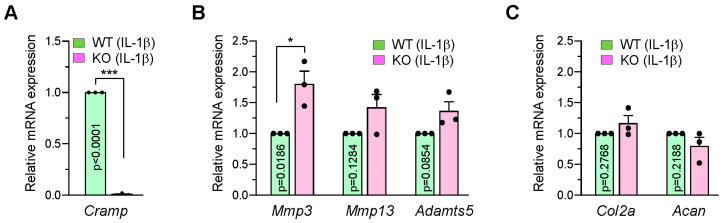
Cramp deficiency does not reduce the mRNA expression levels of catabolic genes in IL-1β-stimulated chondrocytes. Primary cultured chondrocytes from Cramp KO and their WT littermates were treated with IL-1β (1 ng/mL) for 48 h, and relative mRNA levels were determined by real-time RT-PCR. (**A**) Analysis of Cramp mRNA expression in Cramp WT and KO. (**B**,**C**) mRNA expression profiles of catabolic (**B**) and anabolic genes (**C**) in Cramp WT and KO. Values are the mean ± SEM. * *p* < 0.05, *** *p* < 0.001 versus Cramp KO.

The pro-inflammatory cytokine interleukine-1β (IL-1β) upregulates catabolic factors and suppresses anabolic factor expression in chondrocytes [19]. Our prior observation indicated that siRNA-mediated KD of Cramp in chondrocytes reduced IL-1β-induced catabolic gene expression [18]. Therefore, we examined the effect of Cramp depletion on the expression of matrix-degrading enzymes in chondrocytes derived from Cramp KO or WT littermates. Chondrocytes were exposed to IL-1β for 48 h and analyzed for RNA expression (Figure 2A–C). Unexpectedly, Cramp KO chondrocytes demonstrated a slight increase in catabolic gene expression, rather than a decrease. Cramp KO enhanced the mRNA expression of *Mmp3*, but did not significantly alter *Mmp13* and *Adamts5*, or the anabolic genes *Col2a* and *Acan*. Given the findings from the previous siRNA KD experiment [18], these results imply that a minimal level of Cramp might be necessary for modulating catabolic signaling pathways.

### 2.3. Loss of Cramp Accelerates Monosodium Iodoacetate (MIA)-Induced Cartilage Lesions

**Figure 3:**
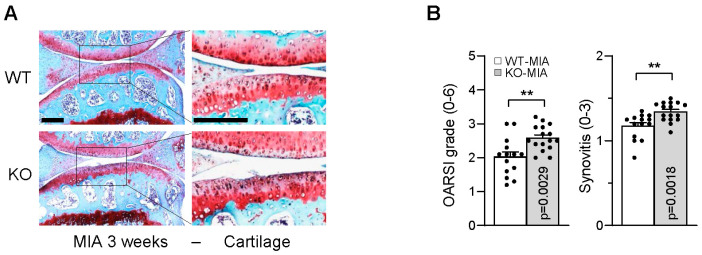
Cramp KO in mouse potentiates monosodium iodoacetate (MIA)-induced cartilage degradation and synovitis. MIA was intra-articularly treated into knee joints of 12-week-old male mice for 3 weeks. (**A**) Representative images of safranin-O staining. Bars = 200 μm. (**B**) Osteoarthritis Research Society International (OARSI) grade and synovitis in Cramp WT (*n* = 14) and KO (*n* = 16). Values are the mean ± SEM. ** *p* < 0.01 versus Cramp WT.

We next examined the in vivo function of Cramp in OA pathogenesis using the MIA-induced experimental OA model. Intra-articular injection of MIA into the knee joint cavity recapitulates OA pathology by inducing acute inflammation and cartilage degradation [20]. Both Cramp WT and KO mice received MIA treatment for 3 weeks and were subsequently analyzed histologically. Notably, safranin-O staining and Osteoarthritis Research Society International (OARSI) scoring demonstrated that Cramp deficiency worsened cartilage tissue degeneration and synovial inflammation (Figure 3A,B). These results suggest a protective role for Cramp in MIA-induced experimental OA.

### 2.4. Genetic Ablation of Cramp Exacerbates the Destabilization of the Medial Meniscus (DMM)-Induced Experimental OA

**Figure 4:**
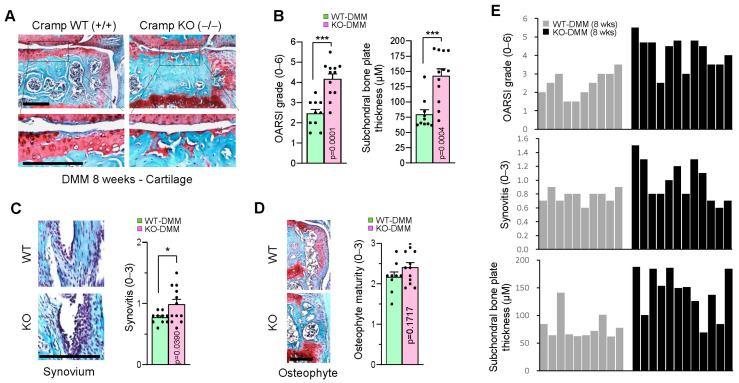
Loss of Cramp exacerbates DMM-induced OA pathogenesis. Thirteen-week-old male mice were subjected to surgical DMM for 8 weeks, and OA-like phenotypes were measured by histological analysis. (**A**) Representative images of safranin-O staining. (**B**) OARSI grade and subchondral bone sclerosis in DMM-operated WT (*n* = 10) and Cramp KO (*n* = 12) are shown. (**C**) Synovial inflammation. (**D**) Osteophyte maturity. (**E**) Correlation between cartilage degeneration and synovial inflammation per mouse in group. Bars = 500 μm. Values are the mean ± SEM. * *p* < 0.05, *** *p* < 0.001 versus Cramp WT.

To further assess whether Cramp deficiency also increases susceptibility to other forms of experimental OA, we performed surgical DMM, a widely used method for inducing post-traumatic OA in mice. Mice underwent DMM surgery for 8 weeks, followed by safranin-O staining of knee joints to evaluate OA-like pathology. In Cramp KO mice, there was greater severity cartilage lesions and subchondral bone sclerosis compared to WT controls (Figure 4A,B). Additional histological analyses showed that loss of Cramp increased synovitis, without significantly affecting osteophyte maturity (Figure 4C,D). The extent of cartilage degeneration in Cramp KO mice appeared to be associated with synovitis but not with subchondral bone sclerosis (Figure 4E). The aggravated OA phenotypes observed in Cramp KO indicate that Cramp deficiency facilitates cartilage destruction in DMM-induced OA. Together, these in vivo findings indicate that Cramp is required for protection against experimental OA.

### 2.5. Cramp Deficiency Increases Chondrocyte Apoptosis in DMM-Induced Experimental OA

**Figure 5:**
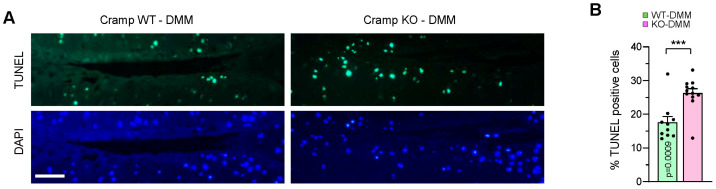
Cramp KO in mice leads to increased chondrocyte apoptosis within the DMM-induced OA model. (**A**) Representative images of cartilage sections subjected to in situ terminal deoxynucleotidyl transferase-mediated dUTP nick end labeling (TUNEL) staining from DMM-operated Cramp WT (*n* = 10) and KO (*n* = 12) mice. DAPI staining was used to detect the number of cell nuclei. Bars = 100 μm. (**B**) Quantification of the number of apoptotic chondrocytes. Values are the mean ± SEM. *** *p* < 0.001 versus Cramp WT.

Chondrocyte apoptosis has been known as a key contributor to cartilage degradation in the context of OA pathogenesis. To elucidate the mechanism underlying OA aggravation in Cramp KO cartilage, we assessed whether loss of Cramp induces chondrocyte apoptosis in the cartilage of DMM-operated knee joints. Notably, Cramp KO mice exhibited a higher number of apoptotic chondrocytes in articular cartilage compared to WT counterparts (Figure 5A,B). These results indicate that Cramp deficiency compromises chondrocyte survival critical for OA pathogenesis.

## 3. Discussion

Accumulating evidence indicates that the Cramp antimicrobial peptide is critically involved in sterile inflammation and tissue degeneration. Cramp exhibits distinct effects on tissue repair depending on the specific tissue type or nature of injury. Cramp has been identified as a protective factor in cases of liver and kidney injuries [14,15]. Conversely, Cramp aggravates muscle degeneration following injury, as well as Duchenne muscular dystrophy [16]. In damaged skeletal muscle, Cramp secreted by immune cells enters myofibers and facilitates muscle degeneration by suppressing the SERCA1 calcium pump. Our prior research suggested a pathogenic function for Cramp in OA. Cramp is highly upregulated in murine OA cartilage, and administration of Cramp in excessive amounts increases the expression of catabolic genes and promotes cartilage degeneration [18]. Nevertheless, this study observed that endogenous and complete Cramp inactivation also results in comparable cartilage destruction. Notably, Cramp deficiency in chondrocytes was associated with an upward trend in catabolic gene expression, even though it did not significantly increase the transcript levels of *Mmp13* and *Adamts5*, which are essential mediators of OA cartilage degradation [4,5]. Additionally, the observed increase in apoptosis among Cramp-deficient chondrocytes raises the possibility that Cramp may serve a protective function in preventing OA cartilage destruction.

In this investigation, we utilized a MIA-induced OA model in Cramp-deficient mice to elucidate the in vivo relevance of Cramp in OA pathology. Unexpectedly, loss of Cramp led to heightened OA-like characteristics, including cartilage lesions and synovial inflammation. Through implementation of surgical DMM, the most widely used OA mouse model, we further demonstrated that Cramp depletion aggravates cartilage degradation, synovitis, and subchondral bone sclerosis in experimental OA. Thus, full inactivation of Cramp seems to exacerbate OA-associated cartilage destruction.

Cathelicidins are known for their immunomodulatory roles, modulating both pro- and anti-inflammatory processes [21,22]. In a mouse model of bacterial meningitis, Cramp deficiency in mice led to a pro-inflammatory phenotype [23,24]. We also observed an elevated synovitis in DMM-operated joints from Cramp KO mice (Figure 4E). Since synovial inflammation plays a role in promoting OA progression [25], increased synovitis in Cramp KO mice could contribute to cartilage degradation. Interestingly, a recent study showed that Cramp derived from immune cells and neurons plays an opposing role in the development of experimental autoimmune encephalomyelitis (EAE), a mouse model of multiple sclerosis [26]. While Cramp produced by neutrophils promotes disease development, Cramp expressed from neurons alleviates disease severity in the late stage of EAE. Therefore, it is possible that Cramp expression in immune cells and chondrocytes differently modulates OA development. To address this hypothesis, future studies employing tissue-specific Cramp KO models will be necessary.

Our past and current findings demonstrate that ectopic Cramp administration accelerates OA pathogenesis by promoting chondrocyte catabolism and meniscal ossification, while complete Cramp deficiency also worsens OA progression by increasing chondrocyte apoptosis. These outcomes collectively indicate that Cramp levels are crucial in determining whether cartilage maintains homeostasis or undergoes degradation. This biphasic effect closely resembles that of NF-κB p65, a central regulator in OA pathogenesis [27]. Specifically, total p65 depletion or high-dose IκB kinase (IKK) inhibition leads to chondrocyte apoptosis and cartilage destruction, whereas p65 haploinsufficiency or low-dose IKK inhibition protects against catabolic activity and OA progression [28,29]. Further studies are needed to characterize the molecular mechanisms that are differentially regulated by Cramp levels. Additionally, it might be interesting to investigate whether Cramp haploinsufficiency or in vivo KD of Cramp using adenovirus-based short hairpin RNA (shRNA) alters cartilage degeneration in OA.

## 4. Materials and Methods

### 4.1. Chondrocyte Culture and RNA Analysis

Mouse primary chondrocytes were isolated from 5-day-old mice via enzymatic digestions as described previously [30]. Briefly, tibial plateaus and femoral condyles dissected from 5-day-old pups were treated with 0.2% collagenase (Sigma-Aldrich, St. Louis, MO, USA; C6885) and 0.01% trypsin/EDTA in serum-free Dulbecco’s Modified Eagle’s Medium (DMEM) (Welgene, Gyeongsan-si, Republic of Korea; LM 001-05) at 37 °C for 2 h, with vortexing every 30 min. After washing with phosphate-buffered saline (PBS), the cartilages were separated using forceps and further treated with 0.2% collagenase in serum-free DMEM at 37 °C for an additional 2 h, again with vortexing every 30 min. Undigested debris was removed using cell strainers, and the isolated cells were counted and seeded into 6-well plates. Cells were cultured in DMEM supplemented with 10% heat-inactivated fetal bovine serum (FBS) and antibiotics at 37 °C in a humidified incubator with 5% CO_2_. Upon reaching approximately 70% confluency, the cells were serum-starved overnight, followed by stimulation with 1 ng/mL of IL-1β (GenScript, Piscataway, NJ, USA; Z02922) for 48 h.

Total RNA was extracted from chondrocytes with TRI reagent (Molecular Research Center, Cincinnati, OH, USA; TR118). First-strand complementary DNA (cDNA) was synthesized using an oligo dT primer and the Improm-II reverse transcription system (Promega, Madison, WI, USA; A3800) in accordance with the manufacturer’s protocol. Quantitative polymerase chain reaction (PCR) analysis was carried out with KAPA SYBR FAST Premix (Kapa Biosystems, Merck, Buchs, Switzerland; KK4621) using a QuantStudio 1 Real-Time PCR System (Applied Biosystems, Foster City, CA, USA). Target gene expression was normalized to *Actb*. The 2^−ΔΔCt^ method was used for data calculation. Primers were synthesized as described in our previous publication [18,30] and a published paper [31]. Primer sequences were as follows: Cramp, 5′-CTACCTGAGCAATGTGCCTTC-3′ and 5′-CAGGCCTACTACTCTGGCTGA-3′; Mmp3, 5′-AGGGATGATGATGCTGGTATGG-3′ and 5′-CCATGTTCTCCAACTGCAAAGG-3′; Mmp13, 5′-TGATGGACCTTCTGGTCTTCTGG-3′ and 5′-CATCCACATGGTTGGGAAGTTCT-3′; Adamts5, 5′-GCCATTGTAATAACCCTGCACC-3′ and 5′-TCAGTCCCATCCGTAACCTTTG-3′; Col2a, 5′-CACACTGGTAAGTGGGGCAAGACCG-3′ and 5′-GGATTGTGTTGTTTCAGGGTTCGGG-3′; Acan, 5′-CTGTCTTTGTCACCCACACATG-3′ and 5′-GAAGACGACATCACCATCCAG-3′; Actb, 5′-AAAGAGAAGCTGTGCTATGTTGC-3′ and 5′-ATGATCTTGATCTTCATGGTGCT-3′.

### 4.2. Mice and Experimental OA

Cramp whole body KO (B6.129X1-Camptm1Rlg/J) mice were obtained from Jackson Laboratory (Bar Harbor, ME, USA; 017799). Animals were housed under specific pathogen-free (SPF) conditions in individually ventilated cage (IVC) racks with access to food and water at the Chosun University animal facility. For the DMM-induced OA model, 13-week-old male Cramp WT and KO mice were anesthetized with isoflurane inhalation and underwent DMM surgery according to a previously described method [32]. Eight weeks post-DMM surgery, tissue samples were collected for histological analysis. For IA injection of MIA (Sigma-Aldrich, St. Louis, MO, USA; I2512), MIA (0.1 mg/10 μL saline) was administered by intra-articular injection into the knee joints of 12-week-old male mice, and samples were collected 3 weeks after MIA administration. Animal procedures were approved by the Chosun University Institutional Animal Care and Use Committee (approval no. CIACUC2022-A0024).

### 4.3. Skeletal Staining, Histological Analysis and In Situ Apoptosis Detection

Mouse embryo skeletal staining was performed to assess skeletal development in whole mouse embryos using alcian blue and alizarin red, following previously described protocols [30,33]. In brief, mouse embryos at embryonic day 18.5 were subjected to skin removal, evisceration, fixation in 95% ethanol for 4 days, and immersion in acetone for 3 days. The specimens were stained using a solution consisting of one volume of acetic acid, one volume of 0.1% alizarin red S in 95% ethanol, one volume of 0.3% alcian blue 8GX in 70% ethanol, and 17 volumes of ethanol for 10 days. Destaining was carried out in 1% KOH for 2 days, followed by incubation in 20% glycerol containing 1% KOH for an additional 14 days.

For paraffin section preparation, knee joints from adult mice that had undergone DMM surgery or MIA injection were fixed in 4% paraformaldehyde (PFA) for 24–48 h, decalcified with 0.5M EDTA for 2–3 weeks, and embedded in paraffin. The paraffin blocks were cut into 5 μM sections. For safranin-O staining, cartilage sections were deparaffinized in xylene, rehydrated in a series of decreasing concentrations of ethanol, and sequentially stained with hematoxylin, fast green, and safranin-O [30]. For the growth plate staining of 2-week-old mice, knee joints were fixed in 4% PFA for 24 h, decalcified with 0.5M EDTA for a week, and embedded in paraffin. Sections were stained using standard methods with either safranin-O/hematoxylin/fast green or alcian blue/hematoxylin/orange G.

Cartilage degeneration was assessed by safranin-O staining and scored according to the Osteoarthritis Research Society International (OARSI) grading system [34]. Scoring was independently conducted by three blinded observers and applied average value. Osteophyte maturity and synovial inflammation scoring followed previously published methods [4,30], and was also scored in a blinded manner by three observers. Subchondral bone sclerosis was evaluated by measuring subchondral bone plate thickness using ImageJ (version 1.53a) software (NIH, Bethesda, MD, USA).

Chondrocyte apoptosis within articular cartilage was identified using the In Situ Cell Detection Kit (Roche Diagnostics, Mannheim, Germany; 11684795910). Briefly, cartilage sections were deparaffinized, rehydrated, and treated with proteinase K (20 μg/mL in 10 mM Tris (pH 7.4) at 37 °C for 30 min) for antigen retrieval. Sections were then stained with reagents according to the manufacturer’s instructions. Nuclei were counterstained with 4′,6-diamidino-2-phenylindole (DAPI). Images were acquired with the EVOS M5000 Imaging System (Thermo Fisher Scientific, Waltham, MA, USA). Quantification involved comparing the number of apoptotic chondrocytes to the total number of DAPI-positive chondrocytes in articular cartilage.

### 4.4. Statistical Analysis

Student’s unpaired two-tailed t-test was conducted for statistical analysis. The number of mice used is represented by n. P values below 0.05 were considered statistically significant.

## 5. Conclusions

In this study, we utilized Cramp KO mice to investigate the in vivo significance of Cramp in OA pathogenesis. Our findings demonstrate that loss of Cramp enhances chondrocyte apoptosis, thereby worsening OA progression. Together with observations from prior reports, this study highlights the pleiotropic function of Cramp via regulation of chondrocyte catabolic activity and apoptosis in OA pathogenesis.

## Data Availability

Data are available from the corresponding author upon reasonable request.
